# Supplementary material for: Identification of Aichivirus in a Pet Rat (Rattus norvegicus) in Italy
Source: Animals (Basel). 2024 Jun 11;14(12):1765. doi: 10.3390/ani14121765 (PMC11200523; doi:10.3390/ani14121765)
Supplement: Supplementary file 1 [file animals-14-01765-s001.zip › animals-2918156-supplementary.pdf]

animals

MDPI

SUPPLEMENTARY MATERIALS

Table S1: Primer pairs and specific probe used for virus detection. The amplified part of the viral genome is indicated in parenthesis.

Virus (target region)		Primers	Bibliography
Human Astrovirus (3'-end region)	Primer forward	5′-CCGAGTAGGATCGAGGGT-3′	Le Cann et al. 2004
	Primer reverse	5′-GCTTCTGATTAAATCAATTTTAA-3′	
	Probe	FAM: 5′-CTTTTCTGTCTCTGTTTAGATTATTTTAATCACC-3′ Tamra	
Aichivirus A and B (capsid gene)	Primer forward	5′-GTCTCCACHGACACYAAYTGGAC-3′	Kitajima et al. 2013
	Primer reverse	5′- GTTGTACATRGCAGCCCAGG-3′	
	Probe	5′-FAM-TTYTCCTTYGTGCGTGC- 3′NFQ (MGB)	
Human Sapovirus (polymerase capsid junction)	Primer forward	5′-GAYCASGCTCTCGCYACCTAC-3′	Varela et al. 2016
	Primer reverse	5′-CCCTCCATYTCAAACACTA-3′	
	Probe	5′- FAM-CCCCTATRAACCA-3′NFQ (MGB)	
Rotavirus (NSP3 gene)	Primer forward	5′-ACCATCTWCACRTRACCCTCTATGAG-3′	Zeng et al. 2008
	Primer reverse	5′-GGTCACATAACGCCCCTATAGC-3′	
	Probe	5′- FAM-AGTTAAAAGCTAACACTGTCAAA-3′(MGB)	
Human Norovirus Genogroup GI (junction ORF1/ORF2)	Primer forward	5′-CGCTGGATGCGNTTCCAT-3′	ISO 15216
	Primer reverse	5′-CCTTAGACGCCATCATCATTTAC-3′	
	Probe	FAM-5′-TGGACAGGAGAYCGCRATCT-3′TAMRA	
Human Norovirus Genogroup GII (junction ORF1/ORF2)	Primer forward	5′-ATGTTCAGRTGGATGAGRTTCTCWGA-3′	ISO 15216
	Primer reverse	5′-TCGACGCCATCTTCATTCACA-3′	
	Probe	FAM-5′-AGCACGTGGGAGGGCGATCG-3′-MGB/NFQ	
Human Hepatitis A Virus (5'-non coding region)	Primer forward	5′-TCACCGCCGTTTGCCTAG-3′	ISO 15216
	Primer reverse	5′-GGAGAGCCCTGGAAGAAAG-3′	
	Probe	FAM-CCTGAACCTGCAGGAATTAA-3′-MGB/NFQ	
Porcine Circovirus 2 and 3 (Capsid gene)	Primer forward	ACGTCCCTTTACTTTCAATTCACA	Kim et al. 2017
	Primer reverse	TATACTTGGTACACACATCCAGAGTCA	
	Probe	FAM-TGAGTTGATTACTGGCACGCCTAAACCAC-BHQ	

Table S2: Exotic companion mammals analyzed: age and sex.

Species	N. Animals	Ages (N, J, A)*	Sex (M, F)
Rats *(Rattus norvegicus)*	21	A	11M + 10F
Mice *(Mus musculus)*	20	A	11M + 9F
Golden hamsters *(Mesocricetus auratus)*	16	J	9M + 7F
Rabbits *(Oryctolagus cuniculus)*	12	N	6M + 6F
Guinea pigs *(Cavia porcellus)*	11	A	6M + 5F
Java squirrels *(Callosciurus notatus)*	6	A	3M + 3F
Mongolian gerbils *(Meriones unguiculatus)*	2	A	1M + 1F
African hedgehogs *(Atelerix albiventris)*	2	A	2M
Sugar glider *(Petaurus breviceps)*	1	A	F
Total	91		

*N: newborn, J: joung, A: adult.

Table S3: List of strains used for construction of phylogenetic tree.

Accession number	Strain	Host	Isolation source	Country	Reference
**AB084788**	Aichivirus B strain:U-1	Bovine	nr	Japan	Yamashita T. et al., 2003
**KJ950942.1**	Norway rat kobuvirus 1 isolate NrKoV-1/NYC-E18b	Rattus norvegicus	feces	USA	Firth C. et al., 2014
**KJ950945.1**	Norway rat kobuvirus 1 isolate NrKoV-1/NYC-A15b	Rattus norvegicus	feces	USA	Firth C. et al., 2014
**KJ950958.1**	Norway rat kobuvirus 1 isolate NrKoV-1/NYC-B9	Rattus norvegicus	feces	USA	Firth C. et al., 2014
**KM977675**	Porcine kobuvirus isolate OH/RV50/2011	Sus scrofa	feces	USA	Liu X.et al., 2015
**KT325852**	Rabbit picornavirus strain Rabbit01/2013/HUN	rabbit	feces	Hungary	Pankovics P et al., 2016
**LC055961**	Kobuvirus cattle/Kagoshima-1-22-KoV/2014/JPN	Bovine	feces	Japan	Otomaru K. Et al., 2016
**LS481160.1**	Aichi virus 1 isolate H496	Human	nr	Spain	nr*
**MF352432.1**	Aichivirus A strain Wencheng-Rt386-2	Rattus tanezumi	feces	China	nr
**MN116647.1**	Aichivirus A strain rat08/rAiA/HUN	Rattus norvegicus	feces	Hungary	Boros A. et al., 2019
**MN336260**	Bovine kobuvirus strain IL35164	Bovine	feces	USA	Wang L. et al., 2020
**MN604700**	Caprine kobuvirus isolate MN1/2018	Goat	feces	USA	Sobhy NM et al., 2020
**MN648601.1**	Rat kobuvirus GZ85	Rattus norvegicus	feces	China	You F.F. et al 2020
**MT180159.1**	Rat kobuvirus GZ488	Rattus norvegicus	feces	China	You F.F. et al 2020
**MT180162.1**	Rat kobuvirus GZ80	Rattus norvegicus	feces	China	nr
**MT180164.1**	Rat kobuvirus MM33	Rattus norvegicus	feces	China	You F.F. et al 2020
**MT180165.1**	Rat kobuvirus GZ85	Rattus norvegicus	feces	China	You F.F. et al 2020
**MW292474.1**	MuKV/YY69/CHN	Rattus norvegicus	feces	China	nr
**MW292475.1**	Murine kobuvirus 1 isolate MuKV/YY19/CHN	Rattus norvegicus	feces	China	nr
**MW292476.1**	Murine kobuvirus 1 isolate MuKV/YN52/CHN	Rattus norvegicus	feces	China	nr
**MW292478.1**	Murine kobuvirus 1 isolate MuKV/XM86/CHN	Rattus losea	feces	China	nr
**MW292479.1**	Murine kobuvirus 1 isolate MuKV/XM34/CHN	Rattus losea	feces	China	nr
**MW292480.1**	Murine kobuvirus 1 isolate MuKV/YN27/CHN	Rattus norvegicus	feces	China	nr
**MW292481.1**	Murine kobuvirus 1 isolate MuKV/XM86/CHN	Rattus losea	feces	China	nr
**NC 001918.1**	Aichi virus, complete genome	Human	feces	Japan	Yamashita T.et al., 1998
**OM049042.1**	Murine kobuvirus 1 isolate SZ-DD6	Rattus tanezumi	feces	China	nr
**OM069744.1**	Murine kobuvirus 1 isolate SZ249	Rattus norvegicus	feces	China	nr
**OM069745.1**	Murine kobuvirus 1 isolate SZ227	Rattus norvegicus	feces	China	nr
**OM069751.1**	Murine kobuvirus 1 isolate SZ188	Rattus norvegicus	feces	China	nr
**OM069755.1**	Murine kobuvirus 1 isolate SZ157	Rattus norvegicus	feces	China	nr
**OM069759.1**	Murine kobuvirus 1 isolate SZ86	Rattus norvegicus	feces	China	nr
**OM069762.1**	Murine kobuvirus 1 isolate SZ22	Rattus norvegicus	feces	China	nr
**ON008386.1**	Feline kobuvirus isolate AHAU-1	Feline	feces	China	nr
**OP659034.1**	Feline kobuvirus strain FKV-21SH050443	Feline	feces	China	nr

*nr: not reported
